# Regional, Artery-Specific Thresholds of Quantitative Myocardial Perfusion by PET Associated with Reduced Myocardial Infarction and Death After Revascularization in Stable Coronary Artery Disease

**DOI:** 10.2967/jnumed.118.211953

**Published:** 2019-03

**Authors:** K. Lance Gould, Nils P. Johnson, Amanda E. Roby, Tung Nguyen, Richard Kirkeeide, Mary Haynie, Dejian Lai, Hongjian Zhu, Monica B. Patel, Richard Smalling, Sal Arain, Prakash Balan, Tom Nguyen, Anthony Estrera, Stefano Sdringola, Mohammad Madjid, Angelo Nascimbene, Pranav Loyalka, Biswajit Kar, Igor Gregoric, Hazem Safi, David McPherson

**Affiliations:** 1 Division of Cardiology, Department of Medicine, McGovern Medical School, Weatherhead PET Center for Preventing and Reversing Atherosclerosis, University of Texas Health Science Center and Memorial Hermann Hospital, Houston, Texas; 2 Department of Biostatistics and Data Science, School of Public Health, University of Texas Health Science Center, Houston Texas; 3 Division of Cardiology, Department of Medicine, McGovern Medial Medical School, University of Texas Health Science Center and Memorial Hermann Hospital, Houston, Texas; 4 Department of Cardiothoracic Vascular Surgery, McGovern Medial School, University of Texas Health Science Center and Memorial Hermann Hospital, Houston, Texas; and; 5 Department of Advanced Cardiopulmonary Therapies and Transplantation, McGovern Medical School, University of Texas Health Science Center at Houston and Memorial Hermann Hospital, Houston, Texas

**Keywords:** quantitative myocardial perfusion, coronary revascularization, cardiac PET

## Abstract

Because randomized coronary revascularization trials in stable coronary artery disease (CAD) have shown no reduced myocardial infarction (MI) or mortality, the threshold of quantitative myocardial perfusion severity was analyzed for association with reduced death, MI, or stroke after revascularization within 90 d after PET. **Methods:** In a prospective long-term cohort of stable CAD, regional, artery-specific, quantitative myocardial perfusion by PET, coronary revascularization within 90 d after PET, and all-cause death, MI, and stroke (DMS) at 9-y follow-up (mean ± SD, 3.0 ± 2.3 y) were analyzed by multivariate Cox regression models and propensity analysis. **Results:** For 3,774 sequential rest–stress PET scans, regional, artery-specific, severely reduced coronary flow capacity (CFC) (coronary flow reserve ≤ 1.27 and stress perfusion ≤ 0.83 cc/min/g) associated with 60% increased hazard ratio for major adverse cardiovascular events and 30% increased hazard of DMS that was significantly reduced by 54% associated with revascularization within 90 d after PET (*P* = 0.0369), compared with moderate or mild CFC, coronary flow reserve, other PET metrics or medical treatment alone. Depending on severity threshold for statistical certainty, up to 19% of this clinical cohort had CFC severity associated with reduced DMS after revascularization. **Conclusion:** CFC by PET provides objective, regional, artery-specific, size–severity physiologic quantification of CAD severity associated with high risk of DMS that is significantly reduced after revascularization within 90 d after PET, an association not seen for moderate to mild perfusion abnormalities or medical treatment alone.

See an invited perspective on this article on page 407.

Cardiac PET remains underutilized despite being the gold standard for quantitative myocardial perfusion to define physiologically severity of coronary artery disease (CAD). Indeed, the current invasive standard for physiologic stenosis severity, fractional flow reserve (FFR), driven by the FAME trial (1), was validated by comparison to quantitative PET (2). Consequently, for a large, prospective, real-world, clinical cohort over long-term follow-up, we asked what artery-specific severity threshold of quantitative perfusion associates with reduced death or myocardial infarction (MI) with and without revascularization in stable CAD.

## MATERIALS AND METHODS

The Weatherhead PET Center for Preventing and Reversing Atherosclerosis, McGovern Medical School, University of Texas Health Science Center at Houston, obtained 5,373 routine diagnostic rest–stress, quantitative, myocardial perfusion PET scans on sequential patients of the authors, referrals by other physicians, and self-referred patients with or at risk of CAD. All subjects signed a written informed consent form for PET and follow-up as approved by the institutional Committee for the Protection of Human Subjects. Complete detailed medical history, all tests, and procedures were obtained at each PET and entered into a dedicated medical record database.

For this study, PET scans were excluded for the following reasons: 374 due to attention-deficit/hyperactivity disorder medications or measured blood caffeine that inhibit vasodilator stress, 312 due to nonstandard stress protocols used in other published research, 40 due to technical failures (0.7%), and 873 due to a long-term event-free follow-up of fewer than 90 d, which is considered too short a follow-up time for meaningful outcomes, leaving 3,774 PET scans for this analysis. As a tertiary care center, our cohort had a high prevalence of CAD and symptoms or multiple risk factors shown in Table 1 for PET groups with and without revascularization within 90 d after PET.

**TABLE 1:** Characteristics of Groups With and Without Revascularization Within 90 Days After PET

	Revascularization				
Characteristic, # and % of group	Yes	SD or %	No	SD or %	*P*
Total (*n* = 3,774)	134	3.60%	3,640	96.40%	
Age (y)	66.2	±9.9	62.0	±11.9	<0.00001
Body mass index	28.5	±4.2	28.0	±4.4	0.173
Male, # and % of total	116	87%	2,694	74%	0.001
Prior PCI, # and % of group	72	54%	1,003	28%	<0.00001
Prior CABG, # and % of group	28	21%	437	12%	0.002
MI in past 3 mo, # and % of group	6	4%	42	1%	0.001
Past MI >3 mo, # and % of group	24	18%	560	15%	0.427
Hypertension, # and % of group	103	77%	2,430	67%	0.014
Dyslipidemia, # and % of group	128	96%	3,253	89%	0.022
Diabetes, # and % of group	35	26%	784	22%	0.206
Past or active smoking, # and %	41	31%	1439	40%	0.037
Medication, # and %					
Statin	115	86%	2,579	71%	0.0001
Antiplatelet	119	89%	2,510	69%	<0.00001
β-blocker	95	71%	1,556	43%	<0.00001
ACEI or ARB	84	63%	1,866	51%	0.009
Calcium channel blocker	24	18%	567	16%	0.465
Diuretic	40	30%	868	24%	0.110
Risk factors only—no history of CAD	36	27%	2,100	58%	<0.00001
Known CAD (MI, angiography, revascularization), # and %	98	73%	1,463	40%	<0.00001
Calcium > 120 HU on CT, # % of group	132	99%	2,748	75%	
Recent typical angina, # and %	59	44%	262	7%	<0.00001
Recent atypical angina, # % of group	4	3%	96	3%	0.8055
Recent typical or atypical angina, # and %	63	47%	358	10%	<0.00001
Angina with vasodilatory stress, # and %	72	54%	257	7%	<0.00001
Stress ST depression > 1 mm, # and %	14	10%	16	0%	<0.00001
Stress EF by ECG gated PET, # and %	61%	12%	70%	10%	<0.00001
Relative rest, % of LV < 60% of maximum – average	7%	±10%	4%	±10%	0.001
Relative stress, % of LV < 60% of maximum – average	28%	±18%	5%	± 10%	<0.00001
CFC severe, % of LV – average	18%	±21%	2%	±8%	<0.00001
CFC severe, % of % LV – median	12%		0%		
Mild CFC > 15% of LV, # and %	116	87%	1,556	43%	<0.00001
Moderate CFC > 15% of LV, # and %	37	28%	222	6%	<0.00001
Severe CFC > 0% of LV, # and %	108	81%	616	17%	<0.00001
Minimum quadrant average CFR	1.436	±0.592	2.39	±0.69	<0.00001
Minimum quadrant average stress flow (cc/min/g)	0.968	±0.463	1.84	±0.66	<0.00001
Minimum quadrant CFR < 2.0, # and %	109	81%	1,055	29%	<0.00001
Global average CFR	1.91	±0.63	2.60	±0.70	<0.00001
Global average stress flow (cc/min/g)	1.36	±0.50	2.05	±0.66	<0.00001
Stress flow maximum (cc/min/g)	2.25	±0.65	2.73	±0.76	<0.00001
CFR maximum	3.20	±0.98	3.65	±1.00	<0.00001

ACEI = angiotensin-converting enzyme inhibitors; ARB = angiotensin receptor blockers; EF = ejection fraction; HU = Hounsfield units.

### Cardiac PET Acquisition

As described previously, subjects were instructed to fast for 4 h and abstain from caffeine and cigarettes for 24 h. Cardiac PET images were acquired using a Discovery ST 16-slice PET/CT scanner (GE Healthcare) in 2-dimensional mode; standard vasodilator stress, primarily dipyridamole, and 1,110–1,850 MBq (30–50 mCi) of ^82^Rb (Bracco Diagnostics); and attenuation correction by cine CT with reduced radiation dose, correct coregistration, and optimal region-of-interest placement for arterial input (3–5).

### Cardiac PET Analysis

Absolute myocardial perfusion in cc/min/g was quantified for each of 1,344 pixels of the left ventricle (LV) images using validated HeartSee software (University of Texas Health Science Center—Houston, Food and Drug Administration [FDA]–approved K171303]) (3–5) with a methodology precision of ±10% (coefficient of variance) on serial rest–rest and stress–stress images in the same patient minutes apart under stable physiologic conditions (5). As requested and expected by referring physicians, every Cardiac PET Consultation Report provides integrated synthesis of all clinical, visual, and quantitative PET metrics as favoring coronary angiography, revascularization for specific coronary artery distributions, or medical management alone depending on clinical judgment of referring physician.

### Coronary Flow Reserve (CFR), Stress Perfusion, and Coronary Flow Capacity (CFC)

**FIGURE 1.**
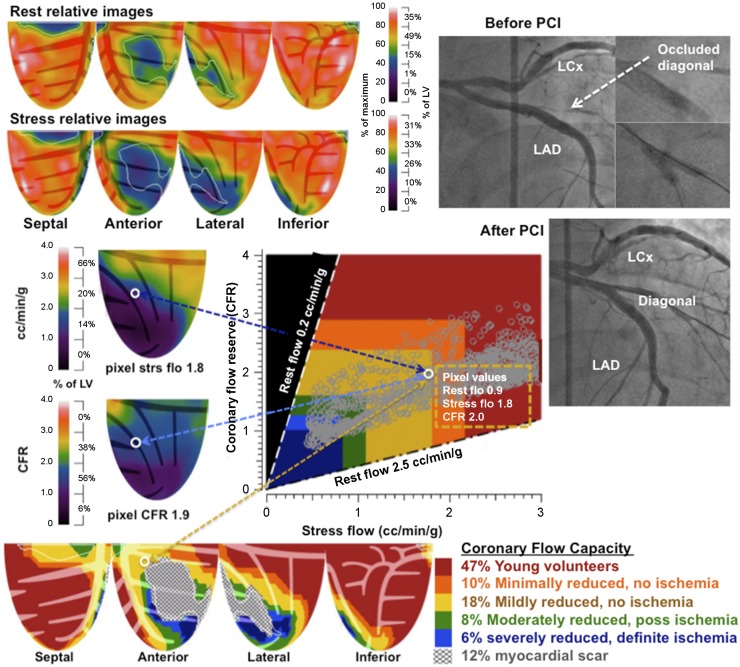
Regional CFC combining pixel values of CFR and myocardial stress perfusion in cc/min/g into simplified integrated size–severity map in specific arterial distribution. Angiogram inset confirms specific secondary artery (diagonal) distribution of quantitative perfusion. Regional quantitative PET resolved problem of recurrent angina after LAD stent for acute MI with cardiac arrest not resolved by initial angiogram as summarized in the text. Color scale bars and histogram to right of each image show color-coded severity and percentage of LV for relative images and for each combined size–severity range of CFC. Dashed white contour on relative images outlines small scar involving 12% of LV (gray hatched area) consisting of transmual scar (blue) comprising 5% of LV in first diagonal distribution, with additional 7% of LV border zones of nontransmural scar (green). This typical target pattern around scar has border zones on relative stress images and CFC map of severely reduced CFC (blue), a next zone of moderately impaired perfusion (green), a next zone of mildly limited perfusion (yellow), and finally normal relative perfusion and CFC (red). To reduce infinite range of CFR and stress perfusion values into 5 objective, clinically relevant categories, CFC map color codes each pixel within 5 color ranges for combined CFR and stress perfusion values of each pixel, spatially maps each pixel back into its LV location with corresponding stress perfusion and CFR values, and calculates percentage of LV for each range of combined both CFR and stress perfusion values listed in CFC color histogram bar for clinically defined groups.

PET perfusion was quantified by automated, objective, size–severity measurements. CFR was computed as stress-to-rest ratio for each of 1,344 pixels. Pixel values of rest–stress relative images, quantitative perfusion, and CFR comprise infinite numbers of values and combinations reflecting true perfusion heterogeneity that require compressing into essential clinically relevant ranges and regional distribution for clinical utility (3–5). Accordingly, the CFC map in Figure 1 color codes each pixel within 5 color ranges for combined CFR and stress perfusion values of each pixel, spatially maps each pixel back into its LV location with corresponding stress perfusion and CFR values, and calculates percentage of LV for each range of combined both CFR and stress perfusion values listed in the CFC color histogram bar.

Figure 1 also illustrates precise, artery-specific, regional size and severity of perfusion abnormalities summarized for this example in the “Results” section. The objective basis for the color-coded CFC plot is detailed in Supplemental Figure 1 (supplemental materials are available at http://jnm.snmjournals.org) as approved by FDA K171303 (3–5), including CFC maps with any pixels coded blue defined as both CFR ≤ 1.27 and stress perfusion ≤ 0.83 that for simplicity is called CFCsevere expressed as percentage of LV.

### Clinical Follow-up

As approved by our Committee For Protection of Human Subjects, prospective programmed follow-up is obtained after every PET scan systematically and continuously by a trained masked research assistant for all-cause death, MI, stroke, first or repeat percutaneous intervention (PCI), or coronary artery bypass grafting (CABG) from clinic or hospital records, mailed questionnaires, phone calls, email, or web searches of newspaper obituaries as an ongoing monthly routine, repeated 3 times for initial nonresponders.

Outcomes are all-cause death alone, combined death/MI/stroke (DMS), and major adverse cardiovascular events (MACE) defined as DMS and revascularization (PCI or CABG), obtained and adjudicated by experienced research assistants, research nurses, and cardiologists masked to PET data. All-cause mortality was analyzed to avoid death misclassification bias.

Stroke was included due to its risk during procedures and its association with the course of atherosclerosis. Revascularization (PCI or CABG) within 90 d after PET was considered as guided by PET. As customary in the literature, fewer than 90 d of event-free follow-up is too short for useful follow-up outcomes after revascularization. Follow-up was obtained for 95% of PET scans at up to 9 y, mean 3.0 ± 2.3 y, with 134 PET scans associated with revascularization (PCI/CABG), 132 associated with death, 56 surviving associated with MI, or 51 associated with stroke.

### Statistical Analysis

We used SAS 9.4 (SAS Institute, Inc.) for multiple Cox regression modeling of covariates of PET metrics plus other clinical characteristics for association with time to first composite MACE (PCI/CABG, DMS) or DMS or all-cause death. We performed colinearity analysis of perfusion measurements before estimation of parameters in final models using clinically guided search of covariates starting with a basic standard risk-factor model for predicting time to first event of MACE, DMS, or death alone.

The second covariate variable group included simple relative objective size and severity of relative perfusion abnormalities as percentage of LV and coronary calcium on CT scan for attenuation correction classified by OsiriX (Pixmeo SARL). The third covariate variable group included all quantitative perfusion metrics. Multiple Cox regression modeling with time-varying covariates was also used to assess effects of revascularization (PCI/CABG) within 90 d after PET as an explanatory variable on subsequent DMS compared with all other clinical and PET characteristics.

Hazard ratios for revascularization versus no revascularization were plotted over time with covariates set to their median values. We used 2-tailed tests and *P* < 0.05 as an indication of statistical significance of rejecting the null hypothesis of null effects. Analysis was performed on a per-scan and per-patient basis with time-varying covariates for outcomes after each PET at different times in the same patient or for different patients.

For significance of discrete variables we used the χ^2^ test, and for continuous variables we used an unpaired *t* test with unequal variance between groups. For comparing survival curves, we plotted Kaplan–Meier plots and used log-rank test. As additional independent covariates for Cox regression modeling, we added the interaction of CFCsevere with revascularization on all-cause death and the propensity score (6) of undergoing PCI/CABG within 90 d that was estimated by logistic regression analysis with the following covariates: male, age, hypertension, diabetes, smoking, dyslipidemia, MI within 3 mo, MI > 3 mo prior, PCI, CABG, coronary calcium, prior abnormal angiogram, stress ejection fraction, resting minimum quadrant average relative severity, vasodilator stress angina or ST > 1 mm depression, clinical angina, taking statin, aspirin, antiplatelet, nitrate, insulin, hypoglycemic, β-blocker, angiotensin-converting enzyme inhibitors/angiotensin receptor blockers, or diuretic medications.

## RESULTS

### Example of Artery-Specific Regional Quantitative PET Perfusion

Since regional precision of quantitative PET may not be widely familiar, Figure 1 illustrates a 59-y-old marathon runner with hyperlipidemia, hypertension, and family history of CAD who had early morning ventricular fibrillation, CPR by his wife, defibrillation by a 911 team, and ST elevation MI with thrombus in the patent left anterior descending coronary artery (LAD) that was stented as the culprit artery. Because of recurrent angina, rest–stress PET was performed, showing a small, severe, nontransmural scar (dark green within an area demarked by dashed white lines) comprising 5% of the LV in the first diagonal distribution.

A border zone of less severe nontransmural scarring (lighter green) comprised another 7% of LV. Average rest perfusion within the rest defect was 0.5 cc/min/g compared with typical 0.25 cc/min/g for transmural scarring. Size and location of the resting scar indicated that the culprit artery for ventricular fibrillation arrest and MI was an occluded first diagonal and not LAD, which was reasonably stented under emergency circumstances as the most accessible without this PET image of the infarcted region in a diagonal distribution or recognition of a flush occlusion of the diagonal branch.

With dipyridamole stress, the rest perfusion defect was larger and more severe, comprising 36% of LV, indicating a large first diagonal branch as the source of angina, whereas LAD distribution showed excellent CFC throughout the septum and apex with no scar. On the basis of PET-quantified extent, severity, and artery-specific location of the original MI and source of recurrent angina, a repeated angiogram showed the culprit subtotal occlusion of the diagonal branch (inset Fig. 1) that was opened with a double balloon procedure (inset) through the mesh of the LAD stent (inset) with resulting patency of the first diagonal (inset) and resolution of angina.

### MACE and Combined CFR and Stress Perfusion (CFC)

**FIGURE 2.**
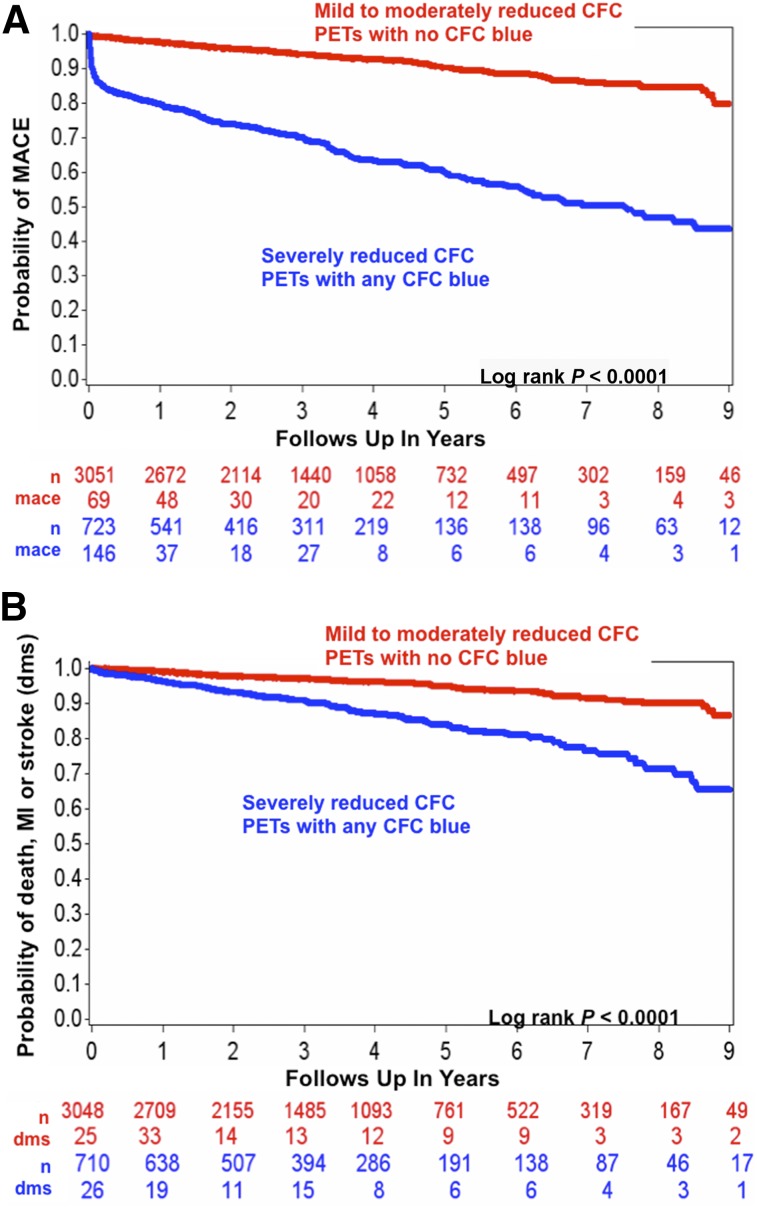
(A) Kaplan–Meier plots of CFC associated with MACE of revascularization, DMS. For CFCsevere, MACE-free survival is substantially lower than for less severe CFC abnormalities. (B) Kaplan–Meier plots of CFC associated with DMS. For CFCsevere, hazard of DMS is substantially higher than less severe CFC abnormalities with increasing difference over time.

Kaplan–Meier plots in Figure 2A show high MACE (death, MI, stroke, PCI, or CABG) associated with CFCsevere defined as pixels having both CFR ≤ 1.27 and stress perfusion ≤ 0.83 cc/min/g (blue) cumulatively expressed as percentage of LV by automated, objective, software. Even small blue regions typically have large moderately severe border zones (green). However, in Figure 2A, PET scans with no severe CFC (no blue) associate with low MACE, a significant difference.

Kaplan–Meier plots in Figure 2B show high risk of DMS associated with CFCsevere (blue) whereas PET scans with no severely reduced CFC (no blue) associated with low risk of DMS, also a significant difference.

In Table 2, multiple Cox regression modeling shows significant association of CFCsevere, revascularization within 90 d after PET, and reduced hazard of DMS by approximately 45% versus no revascularization (*P* = 0.0396). With time-dependent covariates (Supplemental Table 1), DMS was reduced by 54% (*P* = 0.0477). To avoid bias in adjudication of cause of death or MI, Table 3 shows CFCsevere and revascularization within 90 d after PET associated with reduced hazard of all-cause death by 60% compared with no revascularization (*P* = 0.0159).

**TABLE 2:** Multiple Cox Regression Modeling for Association of CFCsevere and DMS After Revascularization Within 90 Days After PET

Parameter	*P*	Hazard ratio	95% hazard ratio confidence limit
Sex (male)	0.3032	1.21	0.842–1.738
Age (y)	<0.0001	1.046	1.03–1.062
Hypertension	0.4178	1.14	0.83–1.568
Diabetes	0.0013	1.627	1.21–2.188
Dyslipidemia	0.4291	0.793	0.446–1.41
MI distant > 3 mo	0.3159	1.195	0.844–1.691
MI recent < 3 mo	0.9883	0.993	0.365–2.698
Prior PCI	0.0069	1.507	1.119–2.028
Prior CABG	0.5423	1.107	0.798–1.535
Coronary calcium	0.2948	1.474	0.713–3.049
Relative stress MQA	0.073	0.984	0.966–1.002
Stress flow MQA	0.0032	1.016	1.005–1.027
CFR MQA	0.7462	0.943	0.663–1.342
CFC severe (blue)	0.0098	0.65	0.469–0.901
PCI/CABG within 90 d	0.0396	0.552	0.313–0.972

MQA = minimum quadrant average for each perfusion metric in the distribution of each coronary artery.

**Table 3:** Multiple Cox Regression Modeling for All-Cause Death After Revascularization Within 90 Days After PET

Parameter	*P*	Hazard ratio	95% hazard ratio confidence limit
Sex (male)	0.0818	1.534	0.947–2.482
Age (y)	<0.0001	1.078	1.055–1.102
Hypertension	0.0684	1.507	0.969–2.342
Diabetes	0.0255	1.566	1.057–2.321
Dyslipidemia	0.3093	0.674	0.316–1.441
MI distant > 3 mo	0.0768	1.491	0.958–2.32
MI recent < 3 mo	0.7581	0.8	0.194–3.303
Prior PCI	0.5199	1.135	0.771–1.671
Prior CABG	0.0313	1.55	1.04–2.311
Coronary calcium	0.9905	1.006	0.4–2.528
Relative stress MQA	0.1042	0.981	0.959–1.004
Stress flow MQA	0.1327	1.01	0.997–1.024
CFR MQA	0.1433	0.667	0.388–1.147
CFC severe (blue)	0.0503	0.621	0.385–1.001
PCI/CABG within 90da	0.0159	0.402	0.192–0.843

MQA = minimum quadrant average for each perfusion metric in the distribution of each coronary artery.

Table 4 shows a significant interaction of CFCsevere and PCI or CABG within 90 d after PET with death (CFCsevere*pcicabg90, *P* = 0.003) thereby validating all-cause mortality as predominantly coronary deaths (Supplemental Fig. 2 provides an additional graph of this interaction). The propensity score and the interaction of CFCsevere with PCI/CABG within 90 d after PET as covariates in the Cox regression model are also shown in Table 4. Table 4 confirms the significantly reduced death in the revascularization group (*P* = 0.036) but also significant residual risk of death (*P* = 0.0007) despite revascularization and significantly more intense medical treatment (Table 1), consistent with more severe, larger perfusion abnormalities and worse risk factors in the revascularized than nonrevascularized group (Table 1).

**TABLE 4:** Multivariable Cox Regression Model for All-Cause Death with Interaction of CFC Severity and Revascularization Within 90 Days After PET with Propensity Score as Covariate

Parameter	*P*	Hazard ratio	95% hazard ratio confidence limit
Sex (male)	0.7392	1.123	0.566–2.23
Age (y)	<0.0001	1.074	1.041–1.107
Hypertension	0.4254	1.285	0.694–2.381
Diabetes	0.0318	1.763	1.051–2.957
Dyslipidemia	0.9919	0.995	0.366–2.707
MI distant > 3 mo	0.4416	1.262	0.698–2.281
MI recent < 3 mo	0.886	1.113	0.258–4.808
Prior PCI	0.5981	0.869	0.516–1.464
Prior CABG	0.2059	1.428	0.822–2.478
Coronary calcium	0.5926	1.739	0.229–13.207
Relative stress MQA	0.5599	0.99	0.959–1.023
Stress flow MQA	0.2598	0.664	0.326–1.354
CFR MQA	0.4168	0.777	0.423–1.429
CFC severe (blue)	0.0194		
PCI/CABG within 90da	0.0355		
CFCsevere*pcicabg90	0.003		
Propensity score	0.0007	12.619	2.891–55.08

MQA = minimum quadrant average for each perfusion metric in the distribution of each coronary artery.

**FIGURE 3.**
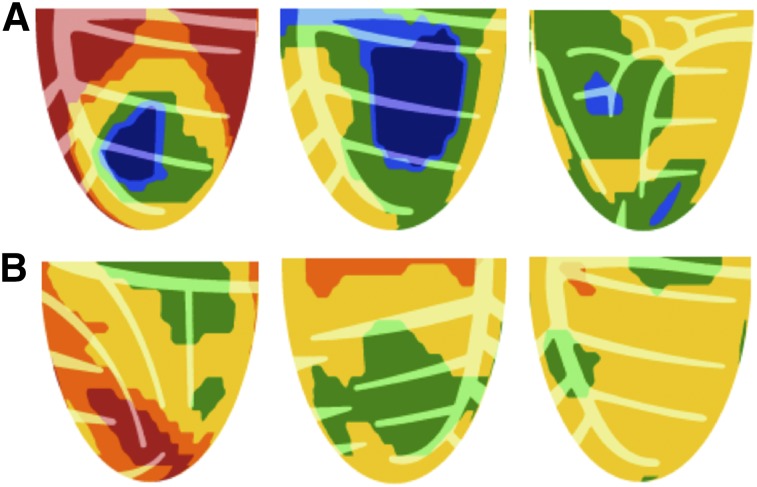
Patterns of severely reduced CFC. (A) Single views of PET scans showing combined CFR ≤ 1.27 and stress perfusion ≤ 0.83 cc/min/g (blue) for greater than zero percentage of LV with surrounding target rings of border zones with less severe CFC. Regardless of different border-zone patterns, severely reduced CFC (blue) associates with high risk of MACE as shown in Figure 2. (B) Single-view examples of mild to moderately reduced CFC (no blue) indicating combined CFR with CFR > 1.27 and stress perfusion > 0.83 cc/min/g, all of which associate with low risk of MACE as shown in Figure 2.

**FIGURE 4.**
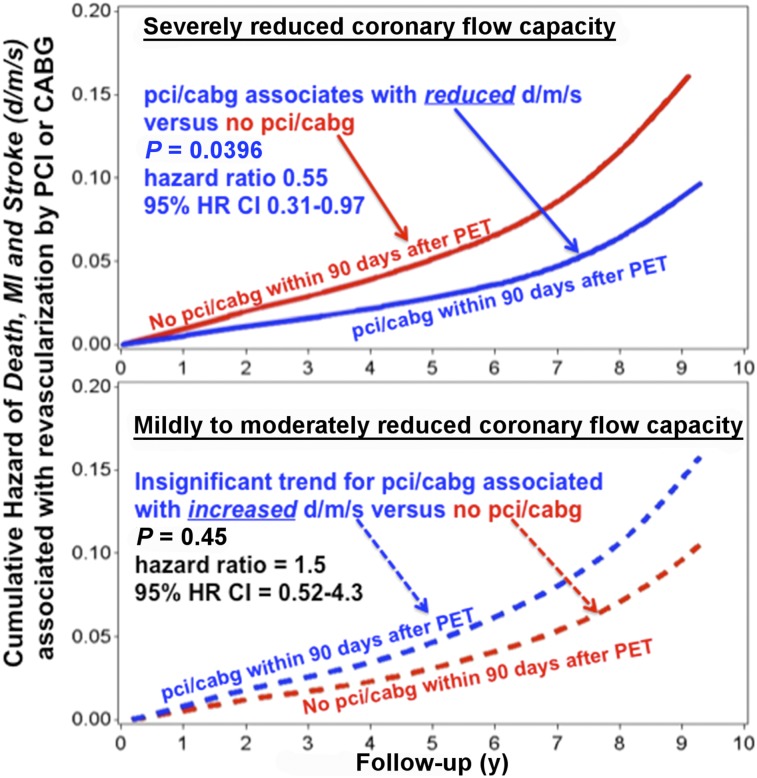
Hazard ratios of DMS (d/m/s) associated with revascularization (pci/cabg) within 90 d after PET (solid blue line) versus no revascularization within 90 d after PET (solid red line) (*P* = 0.0396). For less severe CFC abnormalities consisting of mild or moderate CFC impairment, DMS were insignificantly higher or worse in revascularization (blue dashed line) versus no-revascularization groups within 90 d after PET (red dashed line) (*P* = 0.45).

The single-view PET images of Figure 3 illustrate the range of severe high-risk CFC (blue) and of mild to moderate low-risk CFC (no blue), all with coronary calcification or documented CAD as examples for outcomes in Figures 2 and 4.

Figure 4 displays these outcomes graphically as hazard ratios showing reduced DMS after revascularization within 90 d after PET (solid blue line) versus no revascularization within 90 d after PET (solid red line) (*P* = 0.0396). For less severe CFC abnormalities (no blue) consisting of mild or moderate CFC impairment, DMS were insignificantly higher or worse in the revascularization (blue dashed line) versus no-revascularization groups (red dashed line) (*P* = 0.45).

### PET Metrics Associated with Revascularization and No Revascularization

In Table 1, the PET group with revascularization had significantly worse risk factors, more prior coronary events and procedures, more intense medical treatment, more angina, lower ejection fraction, and substantially more severe, larger relative and quantitative PET perfusion abnormalities with more diffusely reduced PET metrics than the no-revascularization group (for all *P* = 0.0001). For all severely reduced CFC (blue), the histogram of severity–size distribution in the LV was substantially worse in the group with revascularization within 90 d after PET than in the nonrevascularized group by the Kolmogorov–Smirnov statistic (0.39, *P* < 0.0001).

In Table 5, after CFCsevere (blue) was excluded as a covariate in the Cox analysis, other PET metrics alone, including CFCmoderate (green), regional or global CFR, stress perfusion, or relative stress defects, did not associate significantly with reduced DMS after revascularization, reflecting suboptimal selection for benefit from revascularization (Supplemental Tables 2 and 3). Although stress perfusion and CFR alone also failed to associate with reduced DMS, their combination in CFCsevere was significantly associated with decreased DMS after revascularization (*P* = 0.0396, Fig. 3, Tables 2–4) due to CFC accounting for heterogeneity unrelated to stenosis or diffuse CAD severity.

**TABLE 5:** CFCsevere Was the Only PET Metric Significantly Associated with Reduced DMS After Revascularization Within 90 Days After PET by Multivariate Cox Regression Modeling

PET metric	*P*
Coronary flow capacity severe % of LV (blue)	0.0396
Coronary flow capacity moderate % of LV green (no blue)	0.4
Minimum quadrant average stress perfusion cc/min/g (no CFC)	0.32
Minimum quadrant coronary flow reserve (no CFC)	0.08
Global average stress perfusion cc/min/g (no CFC)	0.45
Global coronary flow reserve (no CFC)	0.45
Relative stress defect (% LV < 60% of maximum activity - no CFC)	0.25

In Supplemental Table 4, compared with the revascularization group, the 616 PET scans with CFCsevere not followed by revascularization within 90 d after PET had smaller stress defects and CFC abnormalities, less coronary calcium, higher global and regional CFR and stress perfusion, less clinical angina, less angina or ST depression with PET stress (all with *P* < 0.00001 for Supplemental Table 4), and comparable risk factors. These less severe variables combined with the referring physicians’ clinical decision may explain their lack of revascularization. However, this nonrevascularized group with smaller abnormalities and less angina had higher risk of DMS and all-cause death than the group with more severe, larger perfusion abnormalities undergoing revascularization (*P* = 0.0396; Fig. 4; Tables 2–4).

**FIGURE 5.**
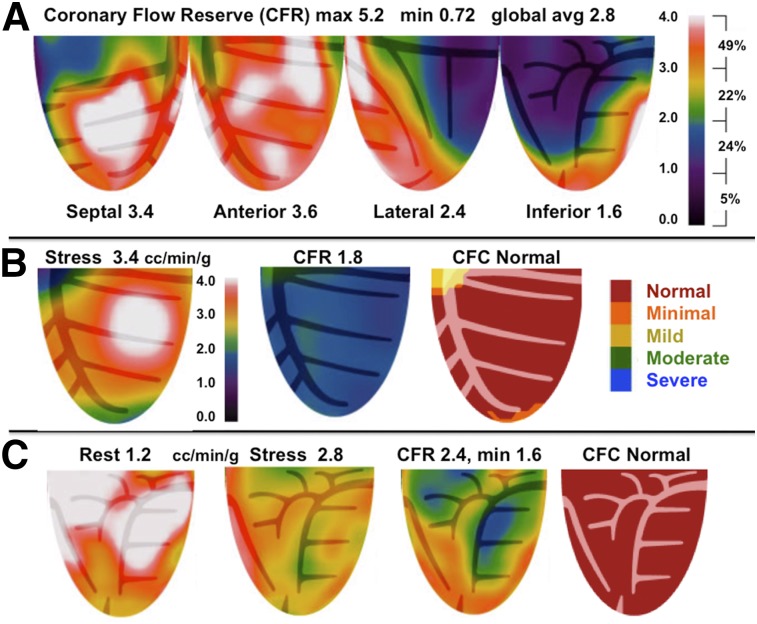
Global perfusion measurements fail to account for severe regional perfusion abnormalities or for resting perfusion heterogeneity with corresponding heterogeneity of CFR. (A) Adequate global CFR of 2.8 that fails to account for severe regionally reduced CFR due to high CFR in rest of LV. Despite adequate global CFR, severely reduced regional CFR associates with high risk of MACE. (B) Single anterior views of high stress perfusion in cc/min/g with low CFR of 1.8 due to high resting perfusion that is accounted for by normal CFC map associated with low CV risk. (C) Single inferior views of heterogeneous high resting and high stress perfusion causing apparent severe regional low CFR alone that is accounted for by low-risk normal CFC map.

The predictive value of regional CFC incorporating regional CFR and regional stress perfusion versus the failure of global CFR or global stress perfusion to associate with reduced DMS after revascularization within 90 d after PET was largely due to regional perfusion heterogeneity (3–5) as illustrated in Figure 5. Three different clinical examples provide clear conceptual physiologic insight on different kinds of heterogeneity commonly seen that explain these statistical outcomes having important clinical impact for personalized artery-specific interventional decisions. As an example, Figure 5A illustrates why global CFR is a poor guide to patient management since global CFR may be good at 2.7, which fails to account for a large, high-risk, severe, stress perfusion abnormality with severely reduced regional CFR that is averaged out in global CFR by high CFR in areas surrounding the severe regional stress defect.

As additional examples, resting perfusion heterogeneity and hence CFR heterogeneity (3–5) may cause apparently low global (Fig. 5B) or regional abnormal CFR (Fig. 5C) due to resting flow heterogeneity but with high global or regional stress perfusion that is accounted for by normal CFC associated with low risk. This CFR heterogeneity due to heterogeneous resting perfusion is so common that it reduces statistical strength of association between CFR alone and decreased DMS after revascularization.

## DISCUSSION

Regional CFC integrating regional CFR and regional absolute stress perfusion in cc/min/g per pixel provide regional, artery-specific, objective size–severity quantification associated with high risk of death, MI and stroke independently of standard risk factors and other quantitative PET metrics. For severely reduced CFC, coronary revascularization within 90 d after PET is associated with reduced hazard of death, MI, and stroke by approximately 50% compared with severely abnormal perfusion without revascularization within 90 d after PET or for revascularization of moderate to mild perfusion abnormalities. The flip side of this data is a corresponding reduction in invasive procedures having no benefit for reducing death or MI in randomized trails.

### Comparison to the Literature

Since the first author originated concepts of CFR for defining physiologic stenosis severity in 1974, pharmacologic stress perfusion imaging in 1978, PET imaging for coronary stenosis in 1978, and FFR in 1993, many reports have been published on these topics. However, this report is the first, to our knowledge, to document regional, artery-specific PET perfusion abnormalities for an objective, quantitative threshold of physiologic severity associated with significantly reduced postrevascularization death, MI, and stroke that is unique for several reasons.

First, global perfusion measurements made in some PET centers fail to account for severe regional stress abnormalities due to high CFR or stress perfusion in surrounding myocardium that average out the regional abnormal perfusion (7). Consequently, the FDA-approved PET software for this study integrates both regional CFR and stress perfusion of each pixel into regional, artery-specific CFC that predicts higher MACE, DMS, death, and their reduction after revascularization than any other single perfusion metric by accounting for regional heterogeneity of resting perfusion and CFR.

Second, others report binary abnormal or normal PET scans using an arbitrary less severe CFR threshold of 2.0 as gatekeeper to a coronary angiogram but without reduced MI or mortality in any randomized trial. In contrast, this study answered a fundamentally different question of what objective, quantitative, high-risk severity of CFC is associated with improved survival after revascularization that is not seen after revascularization of moderate and mild perfusion abnormalities or other PET metrics or compared with medical treatment alone.

The claim that stress perfusion and CFC have no prognostic value over CFR (7) is based on global perfusion that fails to account for even severe regional stress defects as illustrated in Figure 5. That claim is erroneous due to inadequate methodology failing to quantify regional perfusion, thereby explaining the puzzling claim of clinical value based on withholding the quantitative data from referring physicians making clinical decisions (7).

This study had limitations. This study analyzes a nonrandomized, single-center, large clinical cohort by multivariate Cox regression and propensity modeling for adverse outcomes with and without revascularization. Our data provide a scientific basis for PET facilities using other protocols, radionuclides, or scanners documenting comparable reproducibility and severity thresholds for their own PET protocols in cardiology, nuclear medicine, or radiology. Alternatively, PET sites can use the same protocols and FDA-approved software with specific flow models for both ^82^Rb and ^13^N-ammonia with its 3,774 case database for CFC maps accessible on the FDA website.

Our observations suggest that failure of randomized revascularization trials to reduce MI and death may be due in part to lack of objective size–severity quantitative perfusion abnormalities. Future interventional trials may benefit from integrated regional quantitative myocardial perfusion for assessing effects of revascularization on event-free survival. However, it may be difficult to randomize patients with large severe PET defects associated with high mortality or morbidity associated with reduced by revascularization.

## CONCLUSION

CFC integrating regional CFR and regional absolute stress perfusion in cc/min/g by PET provides automated, objective, artery-specific, regional size–severity, physiologic quantification associated with high risk of death and MI that is reduced by 54% after revascularization not seen for revascularization of moderate and mild perfusion abnormalities or for medical treatment alone or for other PET metrics.

## DISCLOSURE

K. Lance Gould received internal funding from the Weatherhead PET Center and is the 510(k) applicant for FDA-approved HeartSee K171303 PET software. To avoid any conflict of interest, K. Lance Gould assigned any royalties arising from PET software to University of Texas for research or student scholarships. Nils P. Johnson received internal funding from the Weatherhead PET Center, has an institutional licensing and consulting agreement with Boston Scientific for the smart minimum FFR algorithm, and received institutional research support from St. Jude Medical (for NCT02184117) and Volcano/Philips Corporation (for NCT02328820), makers of intracoronary pressure and flow sensors. No other potential conflict of interest relevant to this article was reported.

## References

[1] van Nunen LX Zimmermann FM Tonino PAL Fractional flow reserve versus angiography for guidance of PCI in patients with multivessel coronary artery disease (FAME): 5-year follow-up of a randomized controlled trial. Lancet. 2015;386:1853–1860. 26333474 10.1016/S0140-6736(15)00057-4

[2] De Bruyne B Baudhuin T Melin JA Coronary flow reserve calculated from pressure measurements in humans: validation with positron emission tomography. Circulation. 1994;89:1013–1022. 8124786 10.1161/01.cir.89.3.1013

[3] Gould KL Johnson NP Bateman TM Anatomic versus physiologic assessment of coronary artery disease: role of CFR, FFR, and PET imaging in revascularization decision-making. J Am Coll Cardiol. 2013;62:1639–1653. 23954338 10.1016/j.jacc.2013.07.076

[4] Johnson NP Gould KL Integrating noninvasive absolute flow, coronary flow reserve, and ischemic thresholds into a comprehensive map of physiologic severity. JACC Cardiovasc Imaging. 2012;5:430–440. 22498334 10.1016/j.jcmg.2011.12.014

[5] Kitkungvan D Johnson NP Roby AE Patel MB Kirkeeide R Gould KL Routine clinical quantitative rest stress myocardial perfusion for managing coronary artery disease: clinical relevance OT-RV. JACC Cardiovasc Imaging. 2017;10:565–577. 28017383 10.1016/j.jcmg.2016.09.019

[6] Haukoos JS Lewis RJ The propensity score. JAMA. 2015;314:1637–1638. 26501539 10.1001/jama.2015.13480 PMC4866501

[7] Gupta A Taqueti VR van de Hoef TP Integrated non-invasive physiological assessment of coronary circulatory function and impact on cardiovascular mortality in patients with stable coronary artery disease. Circulation. 2017;136:2325–2336. 28864442 10.1161/CIRCULATIONAHA.117.029992 PMC5726898

