# Supplementary material for: Regional, Artery-Specific Thresholds of Quantitative Myocardial Perfusion by PET Associated with Reduced Myocardial Infarction and Death After Revascularization in Stable Coronary Artery Disease
Source: J Nucl Med. 2019 Mar;60(3):410–7. doi: 10.2967/jnumed.118.211953 (PMC6424226; doi:10.2967/jnumed.118.211953)
Supplement: Supplementary file 1 [file jnm211953SupplementalData.pdf]

Detailed methodology for K_i calculation

The measurement of ^{18}F-fluoride metabolic flux (K_i, also referred to as ^{18}F skeletal plasma clearance) provides a more reliable assessment of bone metabolism than standardized uptake values (SUV) in circumstances where bone metabolism averaged across the whole skeleton is sufficiently different from normal that the ^{18}F plasma time-activity curve (TAC) is altered. Examples may include patients with extensive metastatic bone disease (“super scans”), patients with an extensive area of active Paget’s disease, and patients with osteoporosis treated with a potent anabolic bone agent such as teriparatide *(10, 31, 32)*. In such cases the increased avidity of ^{18}F uptake into bone leaves less tracer available for the circulation or for uptake in soft tissue and the ^{18}F concentration in plasma is correspondingly reduced.

The ^{18}F-fluoride semi-population input function (SPIF) was developed so that when combined with a single static PET scan acquired 45 to 90 minutes after injection of tracer it provides a quick and simple method of estimating K_i with little loss of accuracy or precision compared with the conventional 60-minute dynamic PET scan analyzed with an input function generated by continuous arterial sampling (18, 24, 27*)*. An important advantage of this approach is that it enables measurements of K_i to be made at multiple sites in the skeleton at different bed positions with only a single injection of ^{18}F-fluoride tracer.

In the SPIF method the “terminal exponential” is defined as the single-exponential curve fitted to between two and four measurements of ^{18}F venous plasma concentration between 30 and 90 minutes after injection *(4).* At times greater than 30 minutes ^{18}F concentrations in venous and arterial blood are in equilibrium and equal. In the example from the present study shown in Figure S1A, two blood samples were taken at 55 and 85 minutes after injection with plasma concentration measurements of 7.21 and 5.06 kBq/mL after decay correction to the time of injection. The injected activity in this subject was 237 MBq. To generate an estimate of the full plasma TAC between 0 and 90 minutes after injection the terminal exponential calculated from the 55 and 85 minute blood

samples is added to a “residual” curve (Figure S1B) representing the bolus peak and early fast exponentials. The residual curve shown in Figure S1B was produced by averaging data from 10 postmenopausal women who had full arterial blood sampling between 0 and 60 minutes after injection with subtraction of the terminal exponential *(4).* The residual curve in each of the 10 women was normalised to an injected activity of 100 MBq, and the curves averaged after adjusting the time of peak counts to 30 seconds after initiation of the injection protocol. The full SPIF TAC (Figure S1C) was created by multiplying the residual curve by 2.37 (allowing for the injected activity of 237 MBq in this instance) and adding the terminal exponential shown in Figure S1A. To ensure that the contribution from the terminal exponential does not exceed the residual curve in the first 30 seconds after injection the terminal exponential in Figure S1A was rolled off to zero using a ramp function at times before 30 seconds *(24).*

In the example shown in Figure S1C, the terminal exponential accounts for over 80% of the area under the curve between 0 and 90 minutes. The contribution from the terminal exponential exceeds the contribution from the residual curve at times greater than 3 minutes after injection. An important part of the rationale for the SPIF method is that changes in the whole skeleton metabolic flux may alter the terminal exponential, but will have less effect on the residual curve, which is mainly determined by the mixing of the bolus injection throughout the circulation and the rapid early diffusion of ^{18}F-fluoride ions into soft tissue.

In the analysis of conventional 60-minute ^{18}F-fluoride dynamic PET scans, the metabolic flux K_i is often calculated from the bone TAC and the arterial input function using the Hawkins compartmental model (Figure S2) *(11).* However, provided that the rate constant k_4 describing the efflux of tracer out of the bound bone pool is negligibly small, the alternative Patlak analysis method provides a simpler method of calculating K_i from the dynamic scan data. In Patlak analysis the concentration of ^{18}F in the bone region of interest, $C_b(T)$, at time T after injection is expressed by the following equation:

$$\frac{C_b(T)}{C_p(T)} = V + K_i \frac{\int_0^T C_p(t)dt}{C_p(T)} \tag{1}$$

where C_p is the concentration of tracer in plasma and the intercept of the straight line, V, is the volume of distribution in the unbound bone pool (Figure S2). To allow for equilibration between tracer in plasma and the unbound bone pool in the first 10 minutes after injection, the values of K_i and V are determined by fitting a straight line to the 10-60 minute data *(27).*

In practice, the assumption that k_4 is negligibly small is not strictly valid. As a consequence the points of the Patlak plot deviate from the straight-line relationship of Equation 1, and as a result the K_i results underestimate the Hawkins model values. This problem is avoided by using a modified Patlak analysis that introduces a rate constant k_{loss} to represent the efflux of tracer out of the bound bone pool into plasma *(8).* Following the method described by Holden et al. *(33)*, Equation 1 is rewritten as:

$$\frac{C_b(T)}{C_p(T)} = V + K_i\theta(T) \tag{2a}$$

where:

$$\theta(T) = \frac{\int_0^T C_p(t)\exp(-k_{loss}(T-t))\,dt}{C_p(T)} \tag{2b}$$

In this modified analysis the rate constant k_{loss} is varied until the plot of normalized activity $\frac{C_b(T)}{C_p(T)}$ against normalized time $\theta(T)$ from 10-60 minutes after injection gives the best fit to a straight line *(8).* Siddique et al. applied the Holden method to 60-minute dynamic PET scans of the spine and hip and reported mean values of k_{loss} ~0.006 min^{-1} at both sites along with mean values for the volume of distribution V of around 0.2 in the spine and 0.1 in the hip *(23).*

In Siddique's method, the measurement of K_i is made by combining the semi-population input function with measurements of regional bone uptake from a series of short static PET scans acquired at multiple bed positions between 45 and 90 minutes after injection *(23, 27).* The value of K_i is found from a simplified Patlak plot consisting of just two data points, representing the measured tracer

concentration in the bone region of interest at the time *T* of the static scan and the population mean value of the intercept *V.* Rearranging Equation 2a we have:

$$K_i = \frac{\dfrac{C_b(T)}{C_p(T)} - V}{\theta(T)} \qquad (3)$$

K_i is calculated from Equation 3 using the population mean values of *V* and the efflux rate constant k_{loss} *(23,34).*

Acknowledgement

The authors are grateful to Dr Tanuj Puri for creating Supplemental Figure 2.

References

10. Blake GM, Zivanovic MA, McEwan AJ, Ackery DM. ^{89}Sr therapy: strontium kinetics in disseminated carcinoma of the prostate. *Eur J Nucl Med.* 1986;12:447-54.

31. Gnanasegaran G, Moore AE, Blake GM, Vijayanathan S, Clarke SE, Fogelman I. Atypical Paget's disease with quantitative assessment of tracer kinetics. *Clin Nucl Med*. 2007;32:765-9.

32. Blake GM, Siddique M, Frost ML, Moore AE, Fogelman I. Radionuclide studies of bone metabolism: Do bone uptake and bone plasma clearance provide equivalent measurements of bone turnover? *Bone*. 2011;49:537-42.

24. Blake GM, Siddique M, Puri T, et al. A semipopulation input function for quantifying static and dynamic ^{18}F-fluoride PET scans. *Nucl Med Commun*. 2012;33:881-8.

27. Siddique M, Blake GM, Frost ML, et al. Estimation of regional bone metabolism from whole-body ^{18}F-fluoride PET static images. *Eur J Nucl Med Mol Imaging*. 2012;39:337-43.

18. Siddique M, Frost ML, Blake GM, et al. The precision and sensitivity of ^{18}F-fluoride PET for measuring regional bone metabolism: a comparison of quantification methods. *J Nucl Med.* 2011;52:1748-55.

11. Hawkins RA, Choi Y, Huang SC, et al. Evaluation of the skeletal kinetics of fluorine-18-fluoride ion with PET. *J Nucl Med*. 1992;33:633-42.

23. Siddique M, Frost ML, Moore AE, Fogelman I, Blake GM. Correcting ^{18}F-fluoride PET static scan measurements of skeletal plasma clearance for tracer efflux from bone. *Nucl Med Commun*. 2014;35:303-10.

33. Holden JE, Doudet D, Endres CJ, et al. Graphical analysis of 6-fluoro-L-dopa trapping: effect of inhibition of catechol-O-methyltransferase. *J Nucl Med*. 1997; 38:1568-74.

34. Blake GM, Puri T, Siddique M, Frost ML, Moore AEB, Fogelman I. Site specific measurements of bone formation using ^{18}F-sodium fluoride PET/CT. *Quant Imaging Med Surg*. 2018;8:47-59.

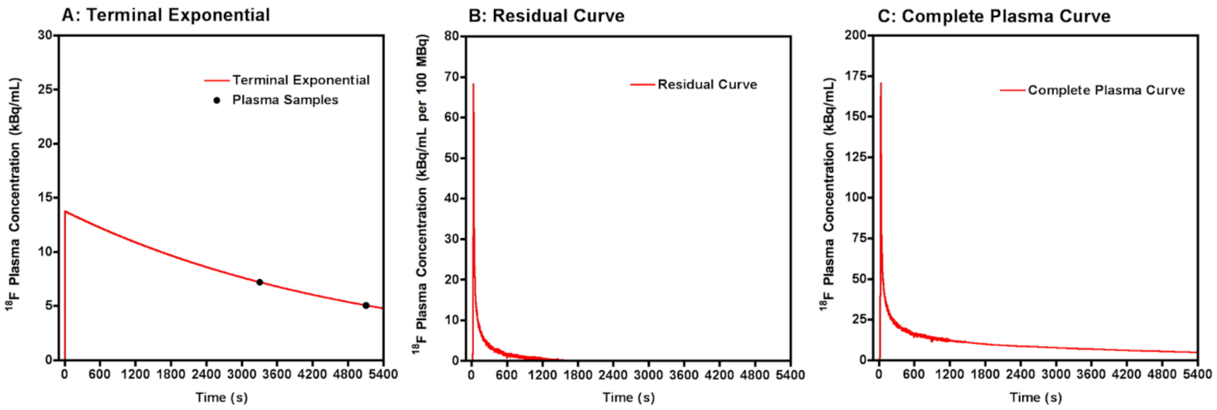

SUPPLEMENTAL FIGURE 1

A) ^{18}F-fluoride plasma concentration curve showing timing of 55 and 85 minute blood samples used to calculate the terminal exponential.

B) Residual ^{18}F-fluoride plasma concentration curve derived from 10 post menopausal women.

C) Resultant full ^{18}F-fluoride plasma concentration curve.

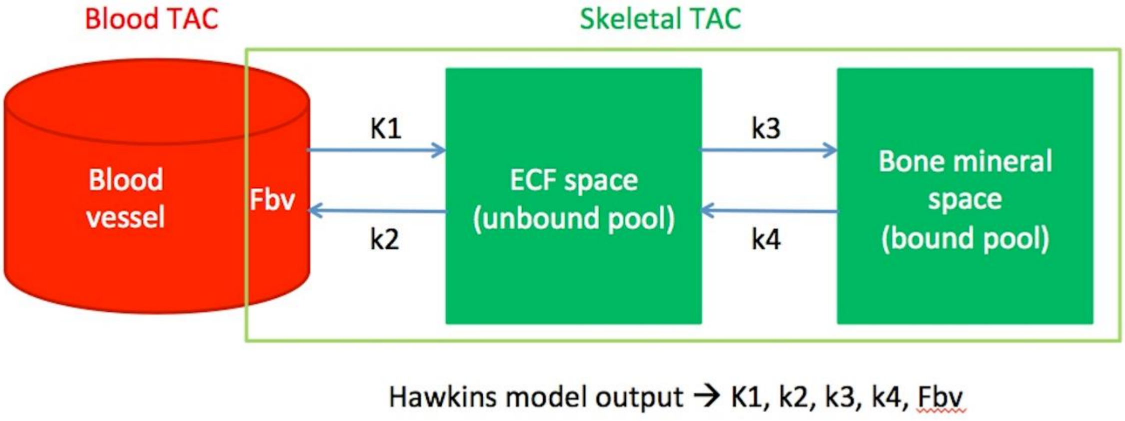

SUPPLEMENTAL FIGURE 2

Illustration of the compartmental model describing ^{18}F-fluoride kinetics in bone

	Lesions (n=52) at baseline				Lesions (n=52) at 8 weeks				% change at 8 weeks			
Patient	Tumor volume (cm^3) baseline	K_i (mL min^{-1} mL^{-1})	SUV_{max} (g/mL)	SUV_{mean} (g/mL)	Tumor volume (cm^3) 8 weeks	K_i (mL min^{-1} mL^{-1})	SUV_{max} (g/mL)	SUV_{mean} (g/mL)	K_i (mL min^{-1} mL^{-1})	SUV_{max} (g/mL)	SUV_{mean} (g/mL)	Clinical reference standard
1	5.9	0.041	28.7	14.7	5.3	0.080	39.9	17.8	95.1	39.0	21.1	PD(CT, BS, ALP,PS)
	4.0	0.029	23.4	10.6	3.8	0.079	42.7	17.7	172.4	82.5	67.0	
	12.9	0.047	42.0	16.9	13.1	0.072	35.8	16.1	53.2	-14.8	-4.7	
	5.2	0.025	16.3	9.1	3.6	0.048	18.8	10.7	92.0	15.3	17.6	
	1.8	0.024	17.8	8.6	1.7	0.099	48.4	22.2	312.5	171.9	158.1	
2	10.3	0.057	32.4	16.3	5.1	0.141	62.2	30.1	147.4	92.0	84.7	PD(CT, BS,ALP, PS)
	2.9	0.076	31.0	21.7	3.7	0.075	24.5	16.0	-1.3	-21.0	-26.3	
	3.2	0.034	18.4	9.9	10.1	0.046	17.4	9.9	35.3	-5.4	0.0	
	5.1	0.100	41.0	28.6	6.9	0.109	30.9	23.4	9.0	-24.6	-18.2	
	12.7	0.085	40.3	24.3	18.7	0.087	28.1	18.6	2.4	-30.3	-23.5	
3	18.1	0.032	30.6	15.0	3.7	0.048	26.9	18.4	50.0	-12.1	22.7	PD(CT, ALP,TM ,PS)
	9.5	0.076	74.0	35.5	11.2	0.192	134.7	73.7	152.6	82.0	107.6	
	6.7	0.040	38.9	18.6	11.7	0.099	85.5	38.1	147.5	119.8	104.8	
	10.3	0.055	60.1	25.5	9.2	0.224	144.3	86.1	307.3	140.1	237.6	
	12.1	0.032	44.3	14.9	18.8	0.046	38.1	17.8	43.8	-14.0	19.5	
4	4.8	0.053	21.7	12.4	1.4	0.080	27.8	16.6	50.9	28.1	33.9	PD(CT, BS, ALP,PS)
	5.5	0.094	34.6	21.9	5.5	0.107	39.1	22.3	13.8	13.0	1.8	
	7.4	0.089	32.9	20.8	8.5	0.103	43.8	21.5	15.7	33.1	3.4	
	3.5	0.088	34.2	20.6	1.7	0.140	52.7	29.2	59.1	54.1	41.7	
	4.4	0.091	30.3	21.2	2.0	0.123	56.6	25.7	35.2	86.8	21.2	
5	4.7	0.068	24.8	11.9	3.3	0.049	18.4	9.8	-27.9	-25.8	-17.6	Non-PD(CT, BS,ALP, TM,PS)
	2.3	0.103	41.3	17.9	2.5	0.110	47.2	21.9	6.8	14.3	22.3	
	4.8	0.029	11.5	5.1	3.2	0.031	11.4	6.3	6.9	-0.9	23.5	
6	11.4	0.172	70.6	40.8	10.6	0.125	48.5	27.5	-27.3	-31.3	-32.6	Non-PD (CT,BS, ALP,PS)
	32.7	0.133	57.3	31.6	25.6	0.168	64.2	37.0	26.3	12.0	17.1	
	6.6	0.140	67.7	33.1	2.5	0.095	35.2	20.9	-32.1	-48.0	-36.9	
	2.8	0.104	41.6	24.7	6.0	0.096	42.1	21.2	-7.7	1.2	-14.2	
	17.9	0.176	67.4	41.8	17.9	0.120	44.6	26.4	-31.8	-33.8	-36.8	
7	5.8	0.094	42.2	26.3	6.7	0.095	29.9	20.7	1.1	-29.1	-21.3	Non-PD(CT, ALP,PS)
	3.0	0.060	27.4	16.9	3.1	0.066	22.1	14.4	10.0	-19.3	-14.8	
	5.6	0.083	38.5	23.2	6.5	0.089	33.9	19.4	7.2	-11.9	-16.4	
	6.2	0.077	43.7	21.6	6.1	0.063	23.0	13.9	-18.2	-47.4	-35.6	

	6.3	0.074	36.3	20.6	5.4	0.069	24.5	15.0	-6.8	-32.5	-27.2	
8	2.9	0.047	44.7	20.5	2.9	0.038	29.0	16.2	-19.1	-35.1	-21.0	Non-PD (CT,BS, ALP,TM ,PS)
	2.5	0.046	37.0	20.3	4.0	0.039	35.1	16.7	-15.2	-5.1	-17.7	
	2.9	0.049	43.7	21.4	5.9	0.036	34.1	15.4	-26.5	-22.0	-28.0	
	2.5	0.051	36.7	22.6	3.0	0.041	33.9	17.6	-19.6	-7.6	-22.1	
	2.3	0.032	31.9	14.0	1.8	0.026	19.6	11.2	-18.8	-38.6	-20.0	
9	2.2	0.062	33.9	17.4	2.8	0.064	32.9	19.0	3.2	-2.9	9.2	Non-PD (CT,BS, PS)
	5.0	0.046	25.9	12.9	3.9	0.051	29.2	15.1	10.9	12.7	17.1	
	1.8	0.081	40.5	22.5	2.2	0.064	34.7	18.9	-21.0	-14.3	-16.0	
	3.4	0.030	15.3	8.6	4.4	0.032	17.0	9.6	6.7	11.1	11.6	
	1.3	0.037	14.7	10.5	2.4	0.047	22.8	14.2	27.0	55.1	35.2	
10	12.7	0.040	25.3	12.4	9.9	0.079	37.5	18.7	97.5	48.2	50.8	Non-PD (CT,BS, ALP,PS)
11	6.3	0.069	29.2	18.6	6.1	0.065	29.7	18.6	-5.8	1.7	0.0	Non-PD (BS,ALP ,TM,PS)
	5.5	0.055	30.9	14.9	5.6	0.088	58.6	25.3	60.0	89.6	69.8	
	1.3	0.040	14.8	11.0	1.3	0.037	15.7	10.9	-7.5	6.1	-0.9	
12	21.4	0.067	45.1	15.3	22.8	0.019	12.4	5.2	-71.6	-72.5	-66.0	Non-PD(CT, BS,ALP, PS)
	10.7	0.049	23.0	11.3	8.4	0.018	14.0	4.9	-63.3	-39.1	-56.6	
	2.8	0.038	12.7	8.8	2.0	0.129	46.8	34.7	239.5	268.5	294.3	
	2.1	0.052	18.2	11.8	2.0	0.055	26.7	15.0	5.8	46.7	27.1	
	8.5	0.088	38.9	20.0	9.2	0.037	18.3	10.1	-58.0	-53.0	-49.5	
Mean	6.8	0.067	35.1	18.8	6.6	0.08	38.3	20.8	35.1	16.0	17.2	

SUV_{mean} (g/ml), SUV_{max} (g/ml) and K_i (mL min^{-1} mL^{-1}) at baseline, 8 weeks, percentage change and mean percentage change at 8 weeks in patients with progressive disease (PD) and non-progressive disease (non-PD)

PS pain score, BS bone scan, CT computed tomography, ALP alkaline phosphatase, TM tumor marker
